# Erratic coil migration in the bronchus after bronchial artery embolization

**DOI:** 10.1002/rcr2.478

**Published:** 2019-08-22

**Authors:** Hideo Ishikawa, Naoki Omachi, Misaki Ryuge, Jun Takafuji, Masahiko Hara

**Affiliations:** ^1^ Haemoptysis and Pulmonary‐Circulation Center Eishinkai Kishiwada Rehabilitation Hospital Kishiwada Japan; ^2^ Center for Community‐based Healthcare Research and Education Shimane University Graduate School of Medicine Izumo Japan

**Keywords:** Bronchial artery embolization, coil, haemoptysis, migration

## Abstract

Herein, we report two cases of erratic coil migration from the bronchial artery to the bronchus after bronchial artery embolization (BAE). Neither patient exhibited haemoptysis recurrence, but chest radiographs revealed that part of the coil had disappeared. In Case 1, the patient coughed up the coil 4.5 years after BAE. We performed repeat BAE to minimize the possibility of haemoptysis considering bronchoscopic and angiographic findings. In Case 2, the patient had severe dry cough 2 years after BAE. Chest radiography showed migrated coils in the trachea; bronchoscopy revealed a migrated fragment of the coil protruding from the elevated mucosa. We used a loop cutter to split the coil and then removed it using forceps. Coil migration to the bronchus is an infrequent late‐stage complication of super‐selective bronchial artery coil embolization, and only one other case has been reported. Accordingly, we propose treatment strategies and speculate on the mechanism of fistula formation.

## Introduction

Bronchial artery embolization (BAE), which is a procedure based on the bronchial artery‐pulmonary artery shunt mechanism, is currently an effective therapy for moderate to severe haemoptysis 1, 2. Various embolic agents are used in BAE, including polyvinyl alcohol, n‐butyl‐2‐cyanoacrylate (NBCA) 1, gelatine sponges, and metallic coils. BAE using coils (super‐selective bronchial artery coil embolization (ssBACE)) is a relatively new method and is regarded safe and effective 2, 3, 4. We reported 1‐year haemoptysis‐free rate, 2‐year haemoptysis‐free rate, and major complications as 90.4%, 85.9%, and 1.6%, respectively, in our long‐term follow‐up of 489 ssBACE cases 2. Erratic coil migration to the bronchus is a very rare complication. To our knowledge, the case described by Morita et al. is the only previously reported case 5. We recently encountered two such coil migration cases. In Case 1, the potential risk of massive haemoptysis and effective method of minimizing this risk were important concerns. In Case 2, the removal of the migrated coil floating in the trachea was the main concern.

The details of these cases are described below. We also discuss the possible mechanism and management of this rare phenomenon.

## Case Reports

### Case 1

A 33‐year‐old man visited our hospital after coughing up a metallic coil‐like substance without any accompanying haemoptysis. He had undergone BAE for cryptogenic haemoptysis 4.5 years previously (Fig. 1A, B). Chest radiography results revealed that a part of an embolized coil placed in the right bronchial artery had disappeared (Fig. 1C). Contrast‐enhanced computed tomography (CT) revealed a small elevated lesion in the left main bronchus several centimetres from the carina (Fig. 2B, C); this lesion was also evident in bronchoscopy (Fig. 2D, E). The lesion was semi‐transparent and vessel‐like, without pulsation. Neither a metallic coil nor an orifice of a fistula was apparent, and only minor bleeding was observed. In the present investigation, bronchial arteriography confirmed that the previously embolized right lower bronchial artery was obstructed. Instead, the right upper bronchial artery was supplying collateral flow to the distal and proximal parts of the coils. The coil‐depleted area between two coils was not enhanced by the contrast medium (Fig. 2D). We suspected that low‐pressure blood flow in the coil‐depleted area was the cause of bleeding in this elevated area. We embolized the right upper bronchial artery to minimize the possibility of haemoptysis from the ruptured right lower bronchial artery (Fig. 1E).

**Figure 1:**
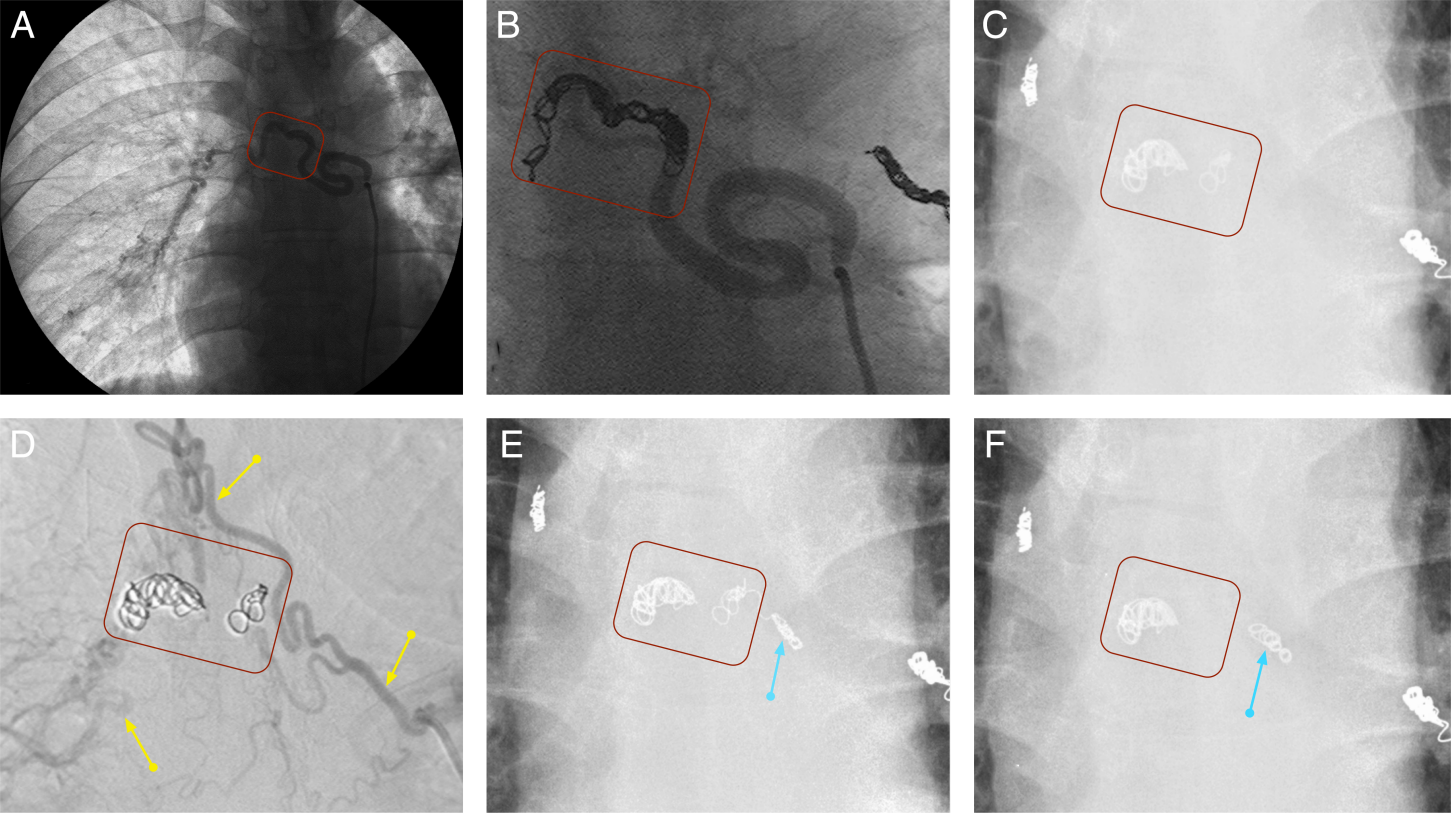
Case 1: Serial changes of the coils. (A) A right lower bronchial arteriography just before the embolization in the first super‐selective bronchial artery coil embolization (ssBACE) session 4.5 years before coughing up coil fragments. The red rectangle shows the position where the coils were going to be installed. (B) A cineangiography result of the first ssBACE of the right lower bronchial artery. The red rectangle shows the coils installed. (C) Chest radiography results after the patient coughed up coil fragments. The red rectangle shows the coils previously placed in the right lower bronchial artery. The depleted part in comparison to (B) was presumed to explain the coughed‐up coils. (D) A cineangiography result of the right upper bronchial artery (yellow arrow). We found that it was supplying collateral blood flow to both distal and proximal parts of the embolized area of the right lower bronchial artery. We suspected that the low‐pressure collateral blood flow was the cause of slight bleeding in the elevated area in the left main bronchus (see Fig. 2D, E). (E) The right upper bronchial artery was embolized. Blue arrow indicates the newly installed coils. (F) Approximately eight months later, the patient revisited our hospital after having coughed up more coil fragments. Chest radiography showed that the coils in the right lower bronchial artery were further reduced (red rectangle). Blue arrow indicates the coils installed in the second ssBACE. They appeared a little looser than (E); however, the coil quantity was presumed unchanged.

**Figure 2:**
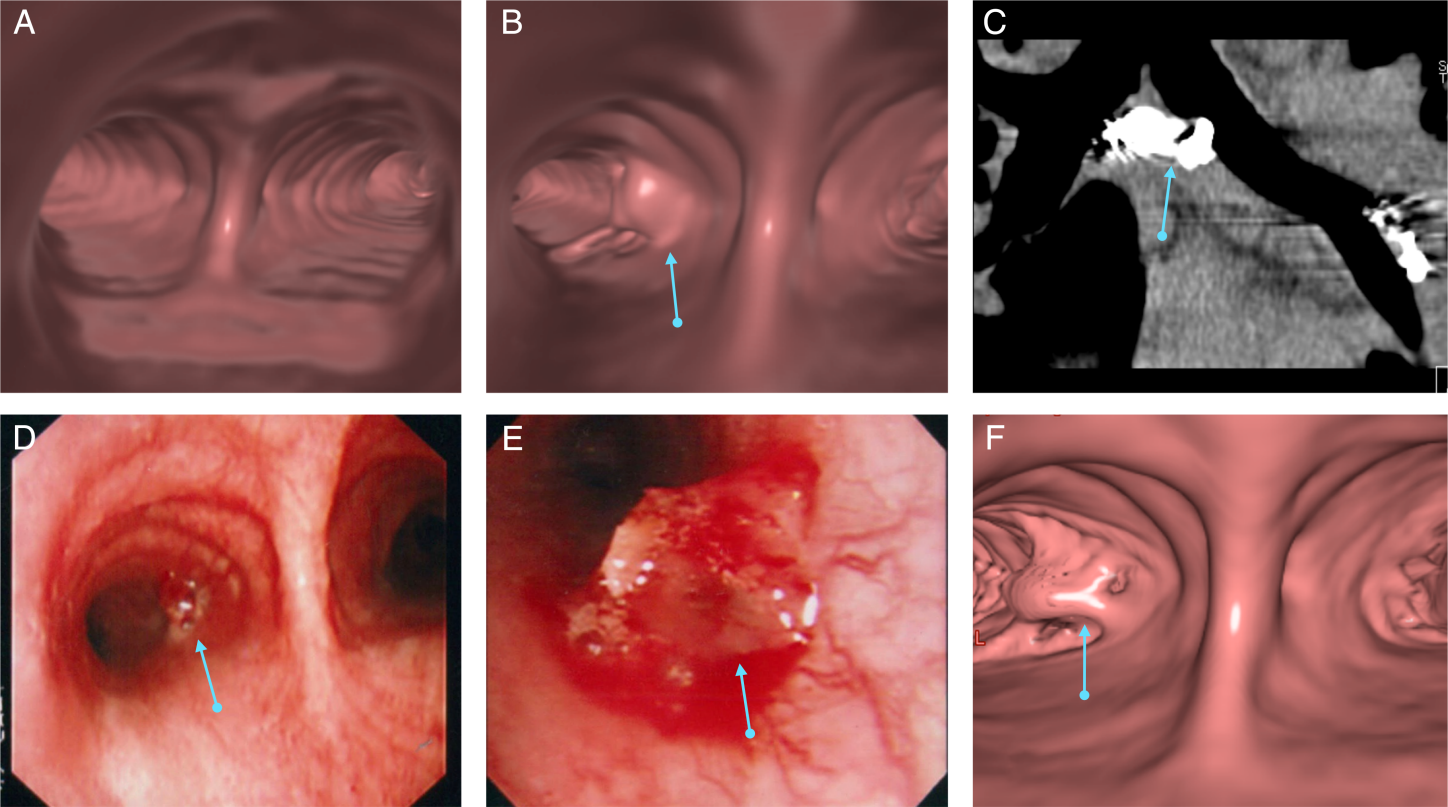
Case 1: Computed tomography and diagnostic bronchography image. (A) A computed tomography images before the first ssBACE. Virtual bronchoscopy around the first carina was normal. (B) Virtual bronchoscopy image after coughing up coil fragments revealed an elevated lesion (blue arrow) in the left main bronchus. (C) A sagittal section around the carina showed that the coils existed just beneath the elevated lesion (blue arrow). (D, E) Diagnostic bronchoscopy results revealed an elevated lesion (blue arrow) in the left main bronchus. We observed slight bleeding around the lesion but could not detect the orifice of the fistula or the coils. (F) Approximately 8 months later, the patient revisited our hospital after having coughed up more coil fragments. Virtual bronchoscopy showed that the small elevated lesion appeared slightly deformed in comparison to the former shape (see Fig. 2B).

Approximately eight months later, the patient revisited our hospital after having coughed up more coil fragments. Chest radiography showed that coils in the right lower bronchial artery were further reduced (Fig. 2G). The small elevated lesion appeared slightly deformed in comparison to the former shape (Fig. 2B, F). He again reported no haemoptysis. Based on clinical findings, we decided that additional ssBACE was not necessary. The coughed‐up coil was presumed to be of the IDC type (Boston Scientific, USA) according to his medical records. He has not complained of haemoptysis for the last 13 years.

### Case 2

A 67‐year‐old woman visited our hospital with a complaint of severe dry cough and throat discomfort. She had undergone ssBACE for cryptogenic haemoptysis 2 years ago (Fig. 3A). Chest radiography and enhanced CT revealed erratic coil migration in the bronchus and the trachea (Fig. 3B, E, F). The coil was located along the internal wall of the bronchus. Bronchoscopy images revealed a migrated fragment of the coil (approximately 7 mm in size) protruding from elevated mucosa at the terminal end of the left main bronchus. The protruding end was moving back and forth, passing through her vocal cord (Fig. 3G–I). We used a loop cutter to split the coil and then removed it using forceps (Fig. 3C). Only minor bleeding was observed, and it stopped quickly during the procedure without the need for any haemostatic measures. Severe dry cough and throat discomfort resolved immediately after the removal of the coil. We recommended that she underwent bronchial artery angiography, and if necessary, ssBACE. However, she refused this procedure considering that there was no recurrence of haemoptysis.

**Figure 3:**
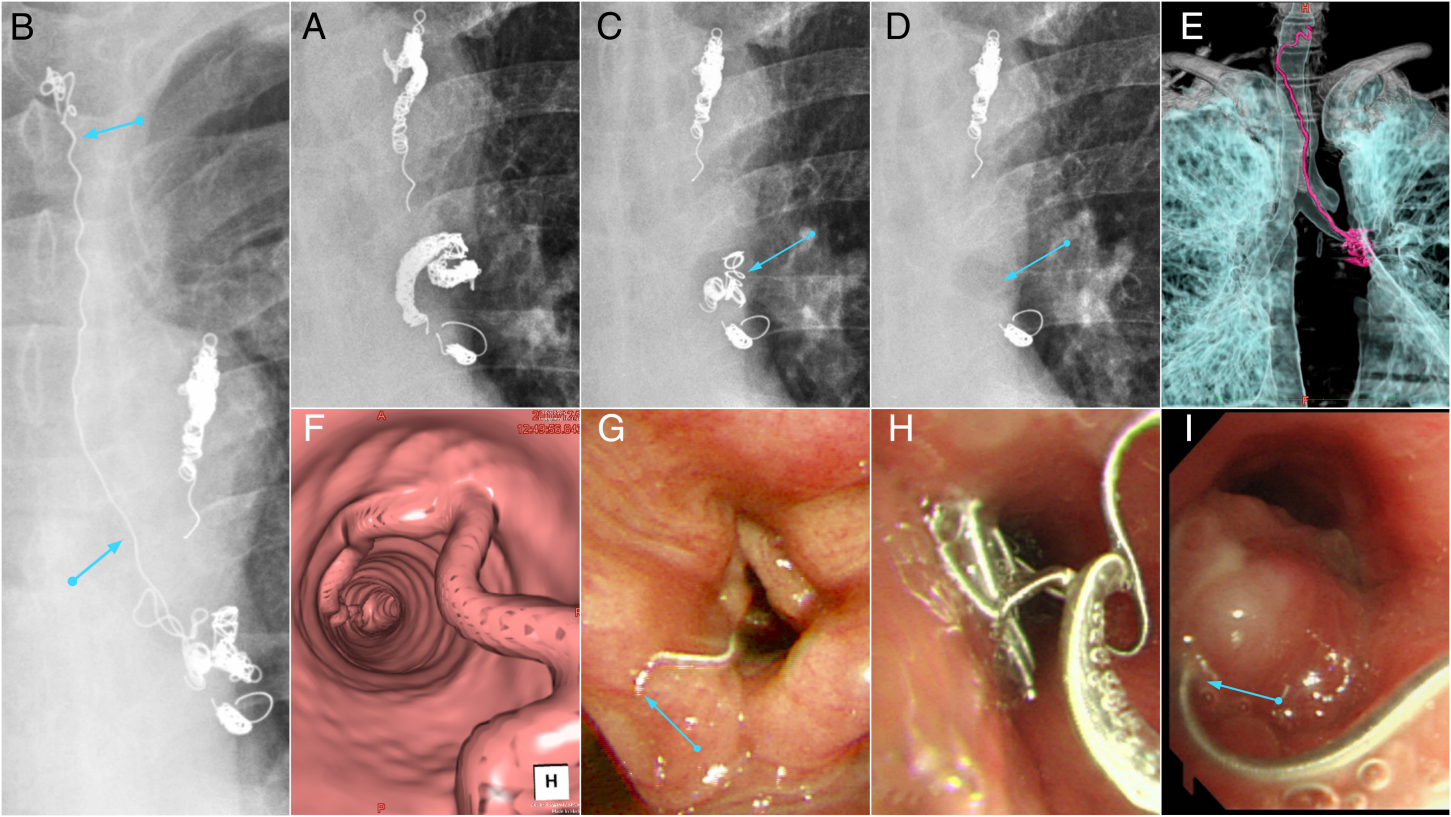
Case 2: (A) Chest radiography results immediately after the first bronchial artery embolization. The left upper and lower bronchial arteries were embolized. (B) Chest radiographs of erratic coil migration. A part of the coils placed in the left bronchial artery was observed in the left main bronchus and the trachea. Blue arrows indicate migrated coils in the trachea and the left main bronchus. (C) We retrieved the migrated coil fragment using a loop cutter and forceps. Blue arrow indicates the residual coils in the bronchial artery. (D) Three months later, the patient revisited us, reporting that she had coughed up a coil fragment. The residual coils observed in (C) disappeared (blue arrow). (E, F) Computed tomography volume‐rendering images of erratic coil migration. Migrated coils are coloured red in (E). (G–I) Bronchoscopy images of the migrated coil. The end of the coil (blue arrow) was moving in and out of the vocal cord (G). This was presumed to be the cause of severe dry cough. In (I) the blue arrow indicates the fistula through which the coil was protruding.

Three months later, she revisited us and reported that she had coughed up a coil fragment. Chest radiography results revealed further reduction of the coil quantity, and no coil parts were observed in the left main bronchus (Fig. 3D). She has not complained of haemoptysis for the past five years.

We inserted two C‐stopper coils (Piolax, Japan) and one Nester embolization coil (Cook Medical, USA) in the perforated area. As determined by microscopic observation, the retrieved fragment was from a C‐stopper coil, not from a Nester coil.

## Discussion

Morita et al. reported the case of a 59‐year‐old woman diagnosed with bronchiectasis who coughed up metallic coils 28 months after ssBACE. She underwent additional ssBACE and then segmentectomy of the right upper posterior lobe (S2); ssBACE was performed to prevent massive haemoptysis through a fistula before and during segmentectomy, which was performed to avoid haemoptysis, infection, and further coil migration through the fistula 5.

In Case 1, we considered the possibility of fatal massive haemoptysis. Bronchoscopy revealed a fistula between the bronchial artery and the bronchus. We detected only minor bleeding around the fistula and surmised that this finding indicated almost complete obstruction of the embolized right lower bronchial artery. Selective right upper bronchial arteriography images revealed new small collateral flow to the embolized right lower bronchial artery, which was presumed to be responsible for the slight bleeding. We performed ssBACE on the right upper bronchial artery. These new coils were deployed in the right upper bronchial artery, which supplied small‐sized collateral flow to the ruptured right lower bronchial artery; therefore, there was little risk of migration of the new coils. Until June 2019, neither haemoptysis recurrence nor infection‐related problems such as mediastinitis had been present for approximately 13 years. This implies that the right lower bronchial artery was completely obstructed.

Case 2 differed from Case 1 and from the case of Morita et al. 5, in that migrated coils were observed in the bronchus and trachea. Bronchoscopic treatment was required to remove the coil, and there was a risk of inducing massive haemoptysis while removing the coil from the bronchus, although no severe bleeding occurred intra‐operatively or post‐operatively. This implies that the ruptured bronchial artery was completely occluded by the initial ssBACE and that there was no blood flow in it.

Morita et al. performed surgery to prevent mediastinitis and further migration of residual coils. We did not follow this procedure, and neither patient developed any infection‐related problems during the later period; this finding suggested that the perforated bronchial arteries were initially completely obstructed and subsequently became organized.

If sufficient blood flow persists in the perforated bronchial artery at the site of fistula formation, severe haemoptysis akin to a Dieulafoy's lesion in the stomach is likely. We investigated the mechanism of haemoptysis recurrence after ssBACE and reported that haemoptysis recurrence was caused by the recanalization of embolized arteries in 45.2% cases, new haemoptysis‐related vessels in 38.5% cases, bridging collateral (where the distal part of an embolized artery receives blood directly from the proximal part of the embolized artery itself) in 14.7% cases, and conventional collateral (where the distal part of an embolized artery receives blood from a different vessel) in 1.7% cases 3. These results suggest that 45.2% of embolized arteries had residual flow, although these data are limited to recurrent haemoptysis cases and cannot be directly extrapolated to the present cases. Nevertheless, if such a recanalized bronchial artery should be perforated, severe massive haemoptysis should not be excluded.

Morita et al. speculated about the reasons for fistula formation. One possibility is the reduced blood flow in the bronchus, thereby leading to bronchial wall necrosis; a second possibility is the existence of a bronchial artery directly beneath the surface of the bronchial wall; a third possibility is fragility of the membranous area of the bronchus; and a fourth possibility is the bronchial artery's long‐term pulsatile pressure through the coils to the bronchial wall. In the present cases, the fistula was not located in the membranous area; thus, the second of the aforementioned possibilities may be the most relevant. Furthermore, the coil brand was not thought to be important, and notably, the brands differed in the present cases.

The ruptured bronchial artery diameters were as large as 5.7 and 6.2 mm in Cases 1 and 2, respectively. In typical cryptogenic haemoptysis cases, haemoptysis‐related bronchial artery's diameter is approximately 1–2 mm. In the case reported by Morita et al., the perforated artery was larger than usual, although its precise size was not mentioned. All the three reported cases were atypical with regard to cryptogenic haemoptysis; therefore, a large vessel diameter may be a risk factor for fistula formation.

If pre‐ssBACE bronchoscopy had been performed in these cases, bronchial Dieulafoy's disease in the bronchus wall might have been detected; however, we did not perform diagnostic bronchoscopy before ssBACE. If pre‐ssBACE bronchoscopy had revealed bronchial Dieulafoy's disease, we could have deployed coils to avoid that point. We, however, do not perform routine pre‐ssBACE bronchoscopy at our institution, and it is not realistic to perform bronchoscopies in all BAE cases. In Case 2, virtual bronchoscopy CT imaging demonstrated a protruded lesion at the fistula area in the bronchus (Fig. 2B). However, we could not detect such a protruded lesion in the same area by pre‐ssBACE virtual bronchoscopy (Fig. 2A); thus, the elevated lesion was formed after the ssBACE procedure.

We have reported two cases of very rare, erratic coil migration from a bronchial artery to a bronchus after ssBACE. Both patients had cryptogenic haemoptysis in which the diameter of the bronchial artery was larger than that observed in typical cases of cryptogenic haemoptysis.

### Disclosure Statement

Appropriate written informed consent was obtained for publication of this case report and accompanying images.
